# Cannabidiol induces autophagy via ERK1/2 activation in neural cells

**DOI:** 10.1038/s41598-021-84879-2

**Published:** 2021-03-08

**Authors:** Talita A. M. Vrechi, Anderson H. F. F. Leão, Ingrid B. M. Morais, Vanessa C. Abílio, Antonio W. Zuardi, Jaime Eduardo C. Hallak, José Alexandre Crippa, Claudia Bincoletto, Rodrigo P. Ureshino, Soraya S. Smaili, Gustavo J. S. Pereira

**Affiliations:** 1 Department of Pharmacology, Escola Paulista de Medicina, Universidade Federal de São Paulo, São Paulo, SP Brazil; 2 Department of Biological Sciences, Diadema Campus, Universidade Federal de São Paulo, Diadema, SP Brazil; 3 Laboratory of Molecular and Translational Endocrinology, Escola Paulista de Medicina, Universidade Federal de São Paulo, São Paulo, SP Brazil; 4 National Institute for Translational Medicine (INCT-TM), National Council for Scientific and Technological Development (CNPq/CAPES/FAPESP), Ribeirão Preto, Brazil; 5 Department of Neuroscience and Behavior, Ribeirão Preto Medical School, Universidade de São Paulo, USP, Ribeirão Preto, Brazil

**Keywords:** Autophagy, Cell death in the nervous system, Diseases of the nervous system

## Abstract

Autophagy is a lysosomal catabolic process essential to cell homeostasis and is related to the neuroprotection of the central nervous system. Cannabidiol (CBD) is a non-psychotropic phytocannabinoid present in *Cannabis sativa*. Many therapeutic actions have been linked to this compound, including autophagy activation. However, the precise underlying molecular mechanisms remain unclear, and the downstream functional significance of these actions has yet to be determined. Here, we investigated CBD-evoked effects on autophagy in human neuroblastoma SH-SY5Y and murine astrocyte cell lines. We found that CBD-induced autophagy was substantially reduced in the presence of CB1, CB2 and TRPV1 receptor antagonists, AM 251, AM 630 and capsazepine, respectively. This result strongly indicates that the activation of these receptors mediates the autophagic flux. Additionally, we demonstrated that CBD activates autophagy through ERK1/2 activation and AKT suppression. Interestingly, CBD-mediated autophagy activation is dependent on the autophagy initiator ULK1, but mTORC1 independent. Thus, it is plausible that a non-canonical pathway is involved. Our findings collectively provide evidence that CBD stimulates autophagy signal transduction via crosstalk between the ERK1/2 and AKT kinases, which represent putative regulators of cell proliferation and survival. Furthermore, our study sheds light on potential therapeutic cannabinoid targets that could be developed for treating neurodegenerative disorders.

## Introduction

Current scientific efforts have focused on describing the broad spectrum of therapeutic effects for cannabidiol (CBD), the major non-psychotomimetic phytocannabinoid derived from the plant *Cannabis sativa*^1^. Extensive studies have shown that CBD has several pharmacological properties, including antidepressant^2^, anti-inflammatory^3^, antiemetic^4^, neuroprotective^5,6^, analgesic^7^, antibacterial^8^, anticonvulsant^9^, anxiolytic^10^, antipsychotic^11^, antitumor^12^ and immunomodulatory activity^13^. However, the underlying mechanisms involved in these properties are still under discussion.

Canonical cannabinoid receptors include CB1 and CB2. The CB1 isoform is highly expressed in the hippocampus, amygdala, basal ganglia, cortex and cerebellum. On the other hand, the CB2 isoform is mainly found in peripheral and immune tissues as well as in microglial and cerebral granule cells^14^. Previous work has shown that endo/exocannabinoids activate G_i/o_ coupled CB1 and CB2 receptors and inhibit adenylyl cyclase^15^, consequently increasing the influx of K^+^ into the cell, resulting in membrane hyperpolarization in the pre-synaptic neurons^16^. Additionally, it has been reported that CBD can modulate the activity of non-canonical receptors, including serotonin 1A receptor (5HT_1A_)^17^, peroxisome proliferator-activated receptor γ (PPARγ)^18^, G-protein coupled receptor 55 (GPR55^19^), μ- and δ-opioid^20^, as well as Transient Receptor Potential Vanilloid 1 (TRPV1) cation channels^21^. It is plausible that this broad range of receptor targets could account for the plethora of CBD-induced pharmacological effects in different tissues. Concerning the TRPV1 receptor, it is a tetrameric protein and a member of the Transient Receptor Potential (TRP) superfamily of nonselective cation channels^22^. Several studies have reported that the TRPV1 receptor is activated by spice ingredients such as capsaicin, low pH, heat (> 43 °C), endovanilloids and endocannabinoids such as *N*-arachidonoylethanolamine (known as anandamide), and/or exocannabinoids such as CBD^21,23–25^.

Despite all the accumulating evidence about the biological properties of cannabinoid compounds, few investigations have characterized its influence on autophagy, and none have fully elucidated the underlying mechanisms behind its action. Autophagy, also referred to as macroautophagy, is a conserved cellular process where the cytoplasmic material (i.e., non-functional organelles and misfolded proteins) is engulfed within a double-membrane vesicle called autophagosome and subsequently degraded and recycled after fusion with a lysosome^26^.

Initially, the microtubule associated protein 1A/1B light chain 3 (LC3), a mammalian homolog of Atg8, located in the autophagosomal inner membrane, plays an essential role in nucleation, elongation and closure of the double-membraned vesicle^27^. It is known that LC3 has two isoforms, LC3-I and II. Under low autophagic stimulation, LC3-I is distributed in the cytoplasm, but following autophagy activation, a phosphatidylethanolamine moiety is added to the protein to form isoform LC3-II that is translocated to the autophagosome membrane^28^. In the final step, LC3-II is inserted into the autophagosome membrane, where it fuses with acidic lysosomes, forms autolysosomes, leads to cargo degradation and ultimately finalizes the autophagic flux^27^.

Notably, Salazar et al. (2009) showed that autophagy mediates CB1-dependent cell death of human glioma is induced by Δ^9^-Tetrahydrocannabinol (Δ^9^-THC), the main psychoactive component of *Cannabis sativa*^29^. The same group also reported that Δ^9^-THC and JMH-015 (a CB2 cannabinoid receptor-selective agonist) induced autophagy and apoptosis in hepatocellular carcinoma via CB2 receptors activation^30^. A study in MDA-MB-231 breast cancer cells reported that CBD induced apoptosis and autophagy in a cannabinoid receptor-independent manner^31^. Recent studies have demonstrated that autophagy dysfunctions and mutations in autophagy-related genes are associated with neurodegenerative diseases^32^. Furthermore, several cannabinoid compounds have been shown to afford protection in different in vitro and in vivo models of neurodegeneration^33,34^.

In the present study, we sought to elucidate the specific CBD-mediated mechanisms involved in autophagy using the human neuroblastoma cell line, SH-SY5Y. Our findings provide evidence that CBD induces autophagy in a concentration-dependent manner that requires crosstalk between the extracellular signal-regulated protein kinases 1 and 2 (ERK1/2) and AKT, also known as Protein kinase B (PKB). These signaling pathways are essential for control proliferation, cell survival and growth^35,36^ and represent a novel target for treating neurodegenerative diseases.

## Results

### CBD regulates cell death in a concentration and time-dependent manner

Previous studies have demonstrated in different cell lines that a relationship exists between autophagy and CBD-mediated cell death^31,37^. To explore this possibility, we used the Propidium Iodide (PI) cell cycle assay to monitor nuclear DNA content in SH-SY5Y cell populations in the presence and absence of CBD and monitored cell death as a function of time. In this assay, the sub-G_0_/G_1_ fraction is an indicator of cell death^38^ .

**Figure 1:**
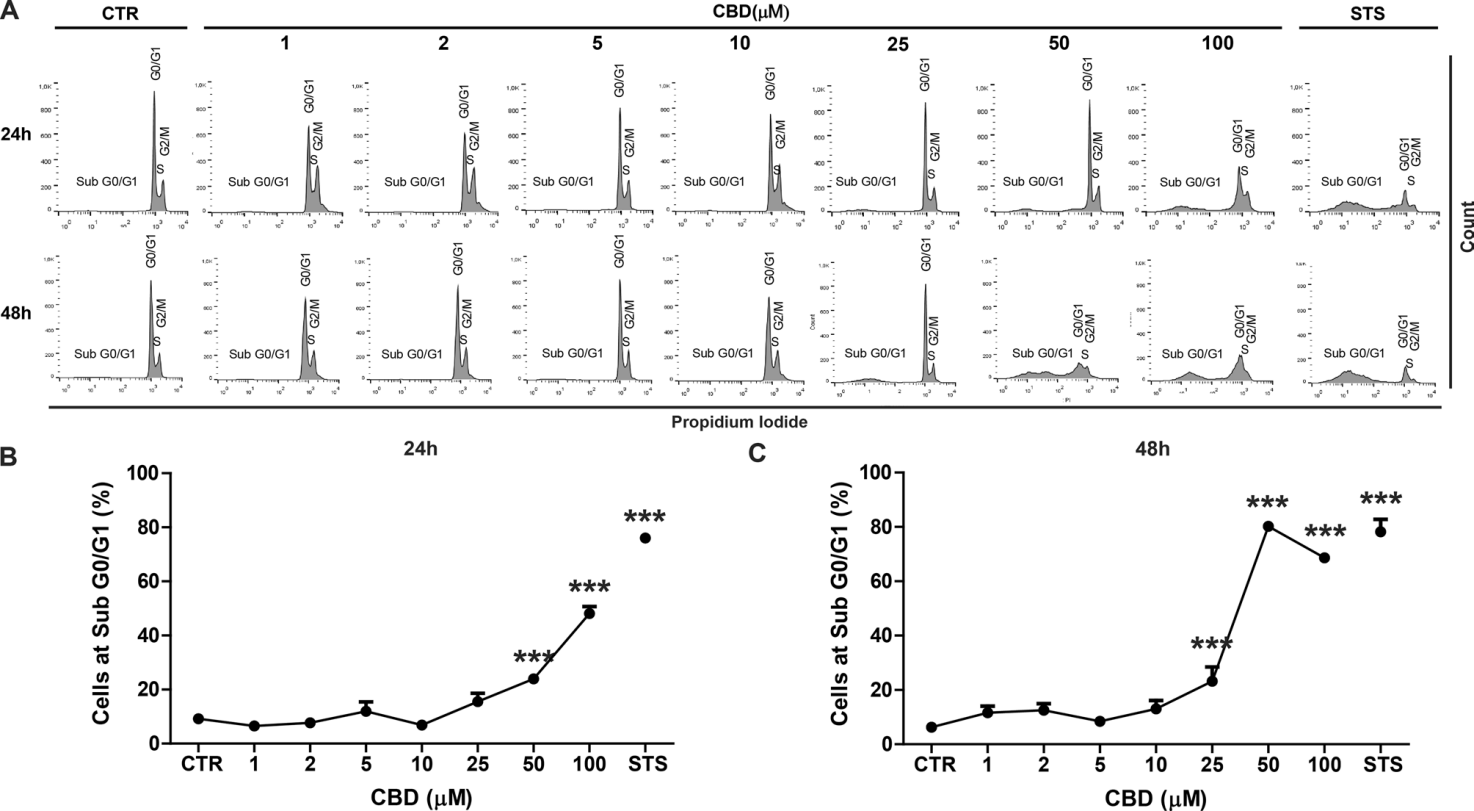
CBD modulates cell death in a concentration and time-dependent manner. SH-SY5Y cells were treated with different CBD concentrations and assessed using the Propidium Iodide cell cycle assay. (**A**) Representative histogram of the sub-G_0/_G_1_ population (an indicative of cell death) of untreated cells (CTR), cells treated with 1, 2, 5, 10, 25, 50 or 100 µM of CBD and 1 µM of staurosporine (STS) for 24 and 48 h. (**B**) The percentage of SH-SY5Y cells in the sub-G_0/_G_1_ phase of the cell cycle after 24 h of treatment with the different CBD concentrations. The results showed that 50 and 100 µM of CBD increased the percentage of cells in the sub-G_0/_G_1_ phase. (**C**) The percentage of SH-SY5Y cells in the sub-G_0/_G_1_ phase of the cell cycle after 48 h of treatment with the different CBD concentrations. The results showed that 25, 50 and 100 µM of CBD increased the percentage of cells in the sub-G_0/_G_1_ phase. Data are expressed as the mean ± S.E.M. Two-way ANOVA (*F*_8,88_ = 22.764) followed by Dunnett’s post-hoc test; ***p < 0.001 relative to the CTR group.

**Figure 2:**
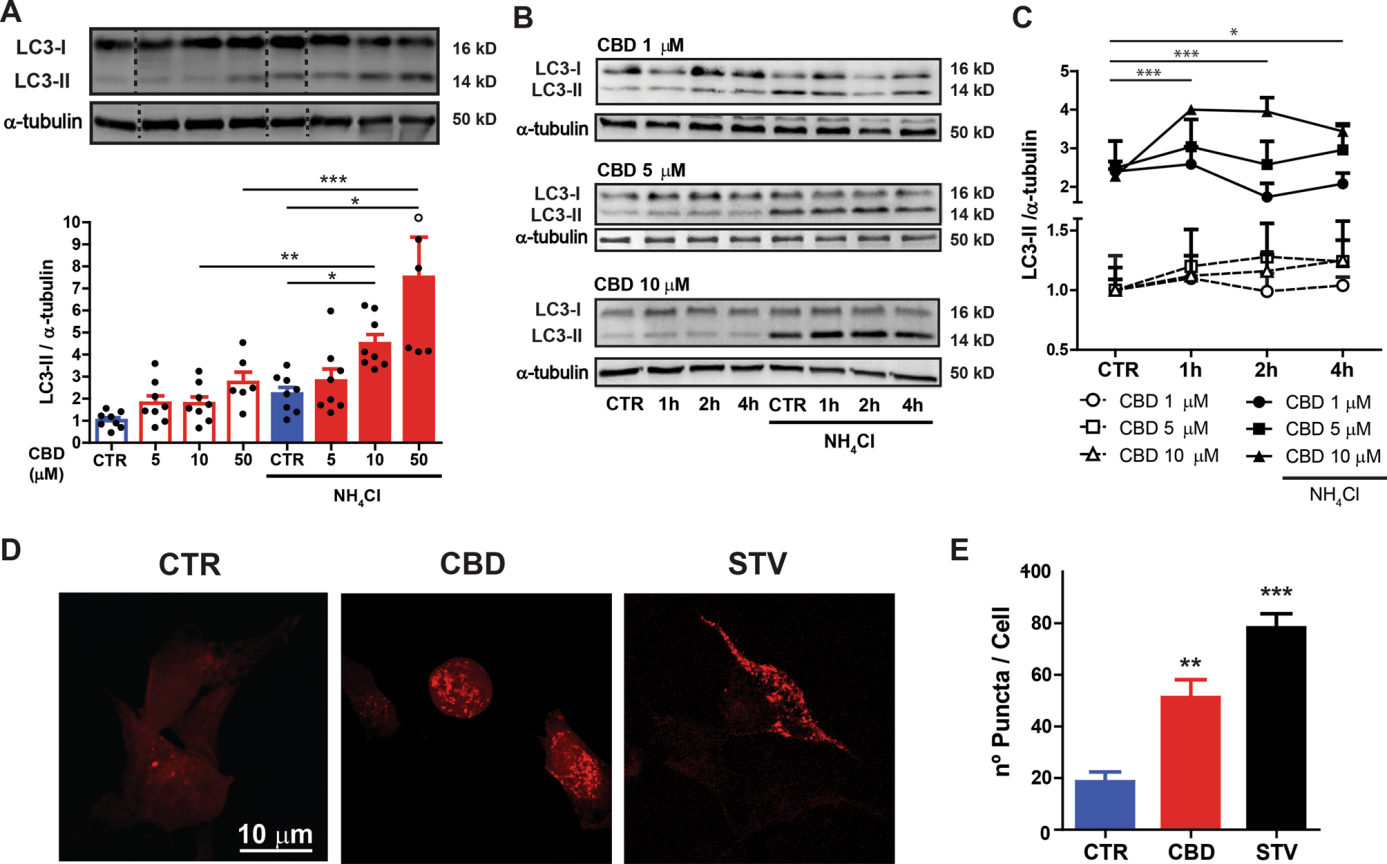
CBD induces autophagy in a concentration-dependent manner. (**A**) The autophagic flux was evaluated in SH-SY5Y cells treated with 5, 10 and 50 µM of CBD for 2 h in the presence or absence of the lysosome inhibitor NH_4_Cl (10 mM) added at the last hour of the treatment. The concentrations of 10 and 50 µM CBD increased LC3-II levels in the NH_4_Cl treated group; Two-way ANOVA (*F*_3,52_ = 3.499) followed by Sidak’s post-hoc test; *p < 0.05, **p < 0.01 and ***p < 0.001. (**B**) Time-course for 1, 5, and 10 µM of CBD on the autophagic flux evaluated at 1, 2 and 4 h in the presence or absence NH_4_Cl added at the last hour of the treatment. The concentration of 10 µM CBD increased LC3-II levels after 1, 2 and 4 h of treatment; Three-way ANOVA (*F*_6,78_ = 3.96) followed by Sidak’s post-hoc test, *p < 0.05 and ***p < 0.001, respectively vs. group treated with NH_4_Cl. Samples were subjected to western blotting using anti-LC3 and anti-α-tubulin primary antibodies. Representative images of LC3-II and a graphical summary reported as the means ± S.E.M of LC3-II levels after α-tubulin normalization are also displayed. (**C**) LC3 puncta count were analyzed in SH-SY5Y cells overexpressing Cherry-LC3 treated with CBD (10 µM) or under starvation conditions (STV) for 2 h. Representative fluorescent images are shown (scale bar, 10 µm). Data are expressed as mean ± S.E.M. One-way ANOVA (*F*_2,22_ = 16.148), followed by Dunnett´s post-hoc test. **p < 0.01 and ***p < 0.001 relative to CTR group. The entire blots are presented in Supplementary Figure S1.

We initially treated SH-SY5Y cells with increasing concentration of CBD (1, 2, 5, 10, 25, 50 and 100 µM), staurosporine (1 µM) or vehicle positive control (H_2_O, DMSO, solvent), conducted the PI flow cytometry assay and analyzed the cell cycle populations after 24 and 48 h of exposure (Fig. 1A–C). Statistical analyses using a two-way ANOVA revealed that CBD significantly increased the sub-G_0_/G_1_ fraction in both concentration- and time-dependent manners [*F*_8,88_ = 22.764; *p* < 0.001]. Concerning concentration dependence, Dunnett’s post-hoc test showed that treating cells with 50 and 100 µM CBD for 24 h increases the number of cells in the sub-G_0_/G_1_ phase (Fig. 1B, p < 0.001). Additionally, concentrations of 25 µM and higher of CBD for 48 h augmented the sub-G_0_/G_1_ fraction (Fig, 1C, *p* < 0.001), thus indicating time-dependent cytotoxicity. Based on these results, subsequent experiments evaluating the autophagic flux were performed by treating the cells with 5, 10 and 50 µM for 2 h (Fig. 2A–C).

### CBD induces autophagic flux in a concentration-dependent manner

While several studies have shown that CBD can potentially modulate autophagy^39–41^, none evaluated whether this drug activates autophagy in a neuronal cell model. For this purpose, we applied different concentrations of CBD (5, 10 and 50 µM) for 2 h in the presence or absence of 10 mM ammonium chloride (NH_4_Cl), a lysosome inhibitor added 1 h before the end of treatment and assessed autophagic flux status. Then we measured LC3-II protein expression, which is indicative of autophagosome accumulation^42,43^. However, the same treatment was not able to modulate the p62 levels (Supplementary Fig. S1E,F).

The data analysis of the LC3-II expression patterns in the presence and absence of the lysosome inhibitor revealed a significant interaction between CBD and NH_4_Cl groups [*F*_3,52_ = 3.499; *p* = 0.022]. Moreover, we found that CBD increased LC3-II levels in a concentration-dependent manner, a result that was further potentiated by NH_4_Cl. These results confirm that CBD treatment modulated autophagic flux of SH-SY5Y cells. Sidak’s post-hoc test showed that 10 and 50 µM CBD under NH_4_Cl treatment increased LC3-II immunoreactivity relative to the control group (untreated cells) both in the presence (*p* < 0.05) and absence (*p* < 0.01) of NH_4_Cl (Fig. 2A).

Next, we determined if the observed CBD-mediated increase in LC3-II expression on autophagic flux was time-dependent. Here SH-SY5Y cells were treated with CBD (1, 5 and 10 µM) for 1, 2 or 4 h, and in some cases, NH_4_Cl was added 1 h before the end of CBD treatment to block autophagy. Analyses of the autophagic flux status, assessed by LC3-II immunoreactivity, indicated interactions between CBD and NH_4_Cl treatment and time interval [*F*_6,78_ = 3.96, *p* = 0.002]. Additionally, Sidak’s post-hoc test showed higher levels of LC3-II levels in cells treated with 10 µM CBD than the respective control group (NH_4_Cl) after 1, 2 (*p* < 0.001) and 4 h (*p* = 0.002) of CBD treatment. Notably, NH_4_Cl potentiated LC3-II levels only in the cells treated with 10 µM CBD, which reached a maximum effect at 1 and 2 h after CBD treatment. Since NH_4_Cl treatment had no additional effect on LC3-II levels with 1 and 5 µM of CBD-treated cells, we can conclude that autophagic flux was not activated with those concentrations (Fig. 2B,C).

To further investigate the ability of CBD to induce autophagy in SH-SY5Y cells, we used a stable cell line overexpressing a fluorescent-tagged mCherry-LC3. These cells were subjected to starvation (STV) in Earle's Balanced Salt Solution (EBSS) and treated with 10 µM CBD for 2 h. The one-way ANOVA showed that CBD induced the formation of a more significant number of intracellular red puncta [*F*_2,22_ = 16.148, *p* < 0.001]. Furthermore, Dunnett’s post-hoc test found that red puncta were significantly more prevalent in the cells treated with CBD (*p* = 0.002) and STV (*p* < 0.001) when compared to the control group (Fig. 2D,E). These data indicate that CBD activated the autophagosome and autolysosome formation.

### The role of CB1, CB2 and TRPV1-receptor activation in CBD-induced autophagy in SH-SY5Y and astrocytes cells

The observation that 10 µM CBD did not reduce cell viability but did promote autophagic flux led us to investigate whether CBD could activate autophagy via canonical cannabinoid receptors. For this purpose, we assessed the autophagic flux in two groups of SH-SY5Y or astrocyte cells. One group of each cell type was treated with 10 µM CBD for 2 h. The other groups of cells were pre-treated (30 min) with CB1, CB2 and TRPV1 antagonists, AM 251, AM 630 and CPZ (10 µM), respectively, in the presence or absence of the lysosome inhibitor NH_4_Cl during the last hour of the treatment.

**Figure 3:**
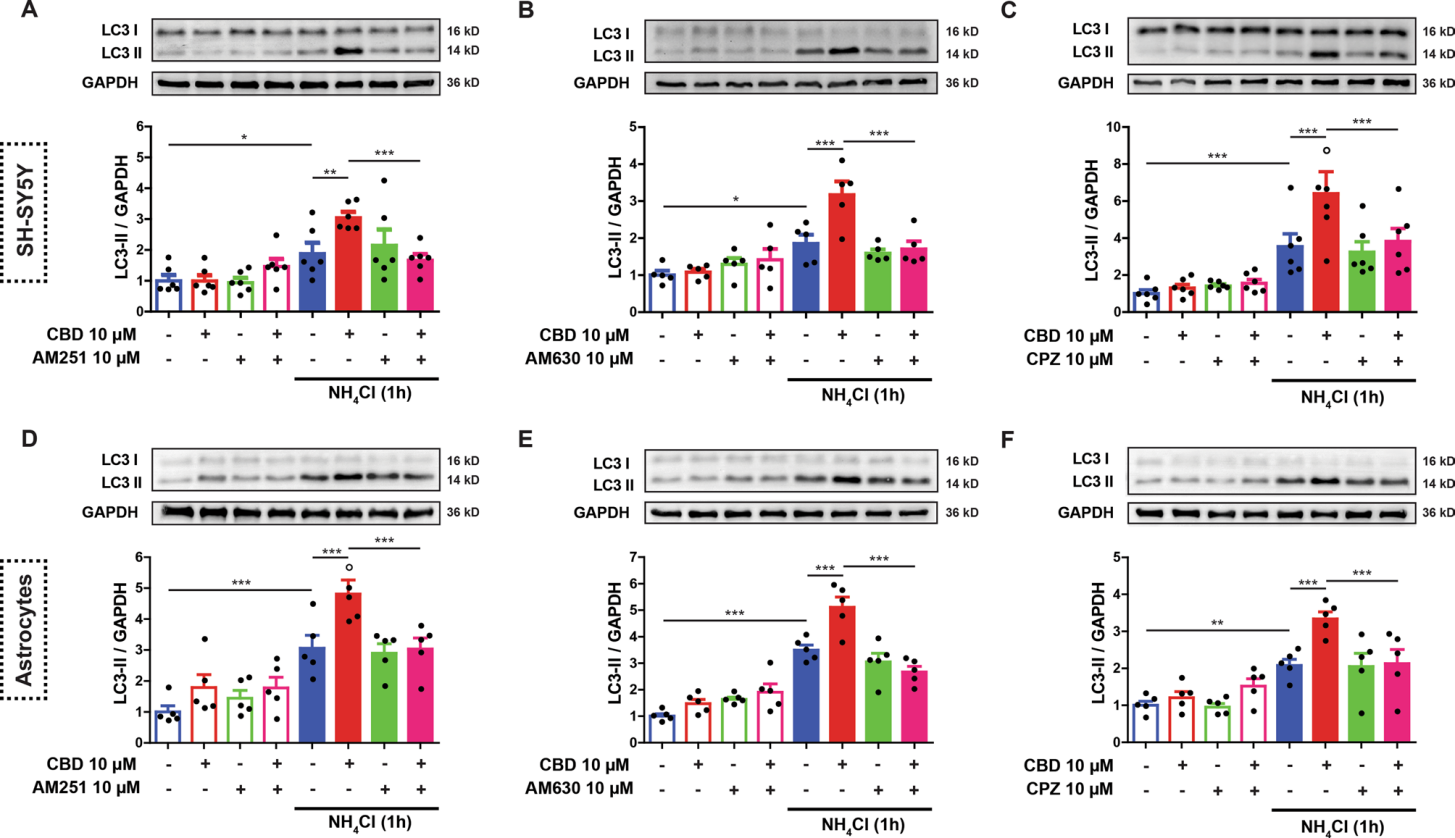
CBD-induced autophagy by CB1, CB2 and TRPV1 receptor activation in neural cells. The autophagic flux was evaluated in SH-SY5Y and murine astrocyte cells after 2 h of treatment with 10 µM CBD and/or with pre-treatment (30 min) with the respective CB1, CB2 and TRPV1 antagonists, AM 251, AM 630 and capsazepine (CPZ) (10 µM each), in the presence or absence of the lysosome inhibitor NH_4_Cl, added during the last hour of treatment. CBD potentiated LC3-II levels under NH_4_Cl blockade, which was reverted by AM 251, AM 630 and CPZ pre-treatment in both SH-SY5Y (A-C) and murine astrocyte (D-F) cells. Samples were subjected to western blotting using anti-LC3 and anti-GAPDH antibodies. Representative images of LC3-II are shown in the panels above the bar graphs. The bar graph data is reported as the means ± S.E.M of LC3-II levels after GAPDH normalization. Analysis was conducted with Three-way ANOVA followed by Sidak’s post-hoc test; *p < 0.05, ** *p* < 0.01 and ***p < 0.001. The statistical summaries for CBD versus antagonist comparisons are detailed in Supplementary Tables S1 and S2. Entire blots are presented in Supplementary Figure S2.

The three-way ANOVA identified a significant interaction for CBD, NH_4_Cl and the respective antagonists in each condition (*p* < 0.05). For example, Sidak’s post-hoc test showed that NH_4_Cl blocked autophagic flux, as evidenced by the augmented levels of LC3-II in the NH_4_Cl treated group relative to the control group (*p* < 0.05). Additionally, CBD treatment consistently increased LC3-II expression (i.e., autophagic flux) under conditions of NH_4_Cl blockade when compared to the respective control groups. Notably, AM 251, AM 630 or CPZ attenuated LC3-II expression to untreated NH_4_Cl-blocked control levels in both SH-SY5Y cells (Fig. 3A–C) and astrocytes (Fig. 3D–F). A summary of the statistics and the respective *F* and *p-values* associated with these experiments are provided in Supplementary Tables S1 and S2. These results provide evidence that CBD-induced autophagy is mediated by the CB1, CB2 and TRPV1 receptors.

### CBD activates autophagy via crosstalk with the ERK1/2 and PI3K/AKT pathways

**Figure 4:**
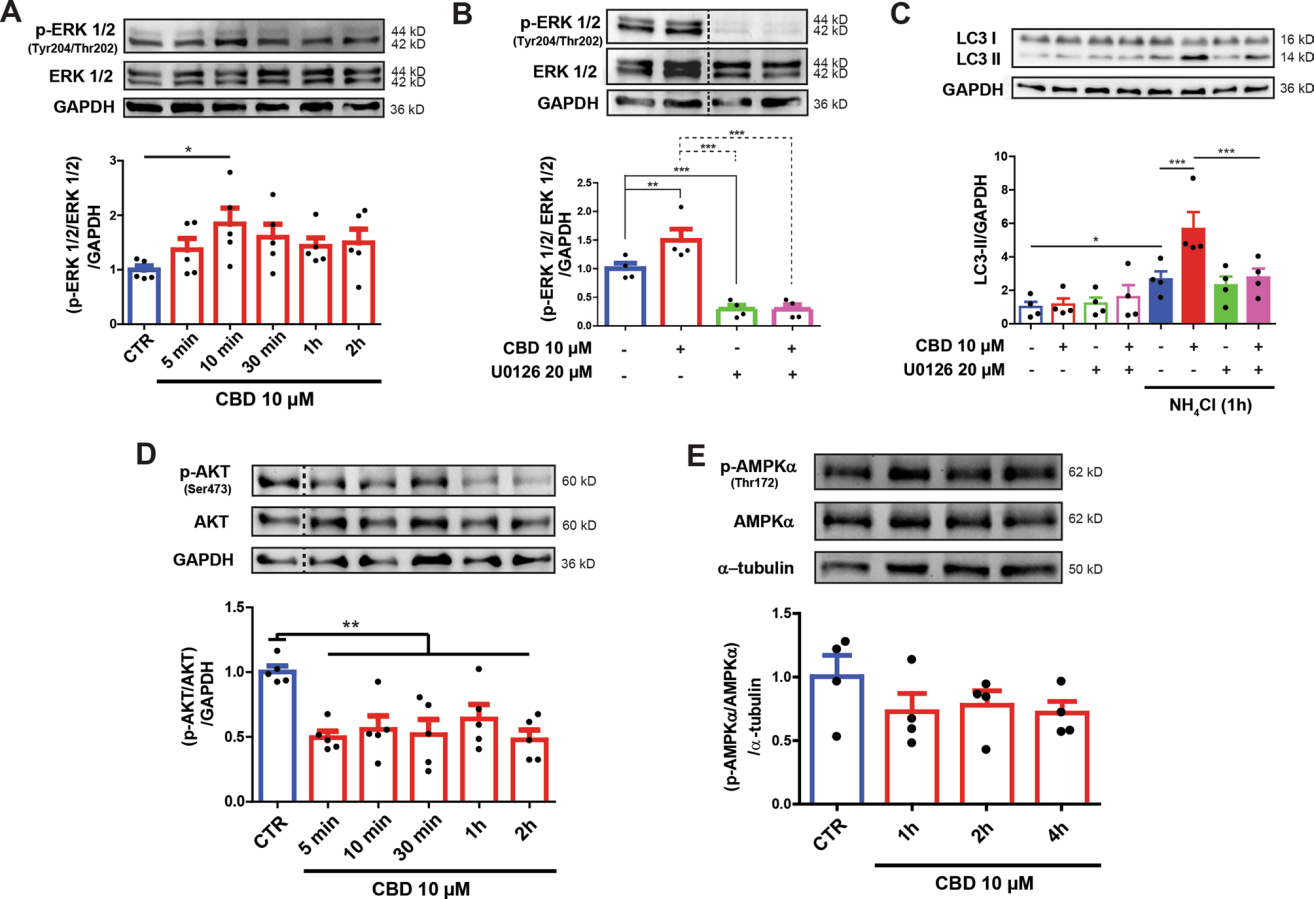
CBD-autophagy is mediated by ERK phosphorylation and AKT dephosphorylation. SH-SY5Y cells were treated with 10 µM CBD for 5, 10, 30 min, 1 and 2 h. (**A**) The membranes were incubated with specific antibodies directed towards phosphorylated kinase ERK1/2 (Tyr204/Thr202) or total ERK1/2 protein. Treatment with 10 µM CBD (10 µM) increased phosphorylated ERK1/2 (p-ERK) protein levels in SH-SY5Y cells after 10 min. (**B**) Cells pre-treated with 20 µM of the ERK1/2 inhibitor U0126 for 30 min and then exposed to 10 µM CBD for 10 min did not exhibit increased levels of phosphorylated ERK1/2 (p-ERK) protein in SH-SY5Y cells. (**C**) Cells were pre-treated with 20 µM U0126 for 30 min and then exposed to 10 µM CBD for up to 2 h, in the presence or absence of the lysosome inhibitor NH_4_Cl, at the last hour of the treatment. The 10 µM CBD treatment increased LC3-II levels under NH_4_Cl treatment, which was reverted with U0126 treatment. (**D**) Total cell lysates from SH-SY5Y cells treated with 10 µM CBD for 5, 10, 30 min, 1 and 2 h. Immunoblots were incubated with a specific antibody for phosphorylated AKT (Ser473) or with an antibody that recognizes total AKT and an antibody against GAPDH. CBD decreased the levels of phosphorylated AKT protein in the SH-SY5Y cells. (**E**) Cells were treated with 10 µM CBD for 1, 2 and 4 h, and the blots were incubated with a specific antibody for phosphorylated AMPK (Thr172) or with an antibody that recognizes total AMPK and an antibody directed against α-tubulin. No effect on AMPK phosphorylation (Thr172) was observed. Analysis was conducted with one, two, or three-way ANOVA followed by Sidak’s post-hoc test; *p < 0.05, ** *p* < 0.01 and ***p < 0.001. Full-length blots are presented in Supplementary Figure S3 and S4.

Having identified receptors that appear to be involved in the CBD-induced autophagy, we then focused attention on the putative signaling pathway(s). Previous studies have shown that the PI3K/AKT and MAPK/ERK pathways regulate cell metabolism, growth, proliferation^35,36^ and participate in autophagy signal transduction^44,45^. Thus, we monitored the phosphorylated and total levels of ERK1/2 (Tyr204/Thr202) and AKT (Ser473) after treating SH-SY5Y cells with 10 µM CBD for 5, 10, 30 min, 1 and 2 h (Fig. 4A). This approach showed that CBD increased ERK1/2 phosphorylation in a time-dependent, bell-shaped fashion. A one-way ANOVA with a polynomial contrast to data regarding p-ERK/total ERK ratio detected a significant effect of the quadratic term on the time interval [*F*_1,24_ = 4.307; *p* = 0.049]. Furthermore, Dunnett’s post-hoc test found that 10 µM CBD significantly increased the p-ERK1/2/total ERK1/2 ratio after only 10 min of treatment (*p* = 0.04). It should be pointed out that this value returned to control levels after 2 h.

Next, we investigated whether CBD-mediated ERK1/2 activation could induce autophagy in SH-SY5Y cells. In this experiment, untreated and CBD-treated cells were pre-treated with 20 µM of the Mitogen-Activated Protein Kinase (MAPK) kinase 1/2 inhibitor (MEK1/2), U0126, for 30 min and assessed CBD autophagic flux^46^. The two-way ANOVA showed that U0126 effectively inhibited CBD-induced ERK phosphorylation [*F*_1,12_ = 7.120; p = 0.017] (Fig. 4B). Additionally, the three-way ANOVA showed that U0126 pre-treatment abolished the CBD-induced increase in LC3-II levels [*F*_1,24_ = 4.763 p = 0.037] (Fig. 4C). These results confirm that ERK1/2 signaling is an upstream regulator of CBD-mediated autophagy.

Similar experiments were also utilized to evaluate the p-AKT/total AKT ratio for up to 2 h after CBD. The one-way ANOVA highlighted a significant effect for CBD treatment [*F*_5,24_ = 5.236; *p* = 0.002]. The Dunnett’s post-hoc test showed that CBD treatment led to a sustained reduction in p-AKT (Ser473) levels during the entire experimental period (*p* < 0.01) (Fig. 4D).

Lastly, we evaluated the effects of CBD on AMP-activated protein kinase α (AMPKα) phosphorylation at Thr172 after 1, 2 and 4 h. While there was a tendency for the p-AMPKα/total AMPKα ratio to appear reduced at all of the time points, a one-way ANOVA failed to detect any significant difference compared to the control group [*F*_3,12_ = 0.537; *p* = 0.670]. Thus, CBD does not appear to play a role in regulating AMPKα signaling (Fig. 4E).

**Figure 5:**
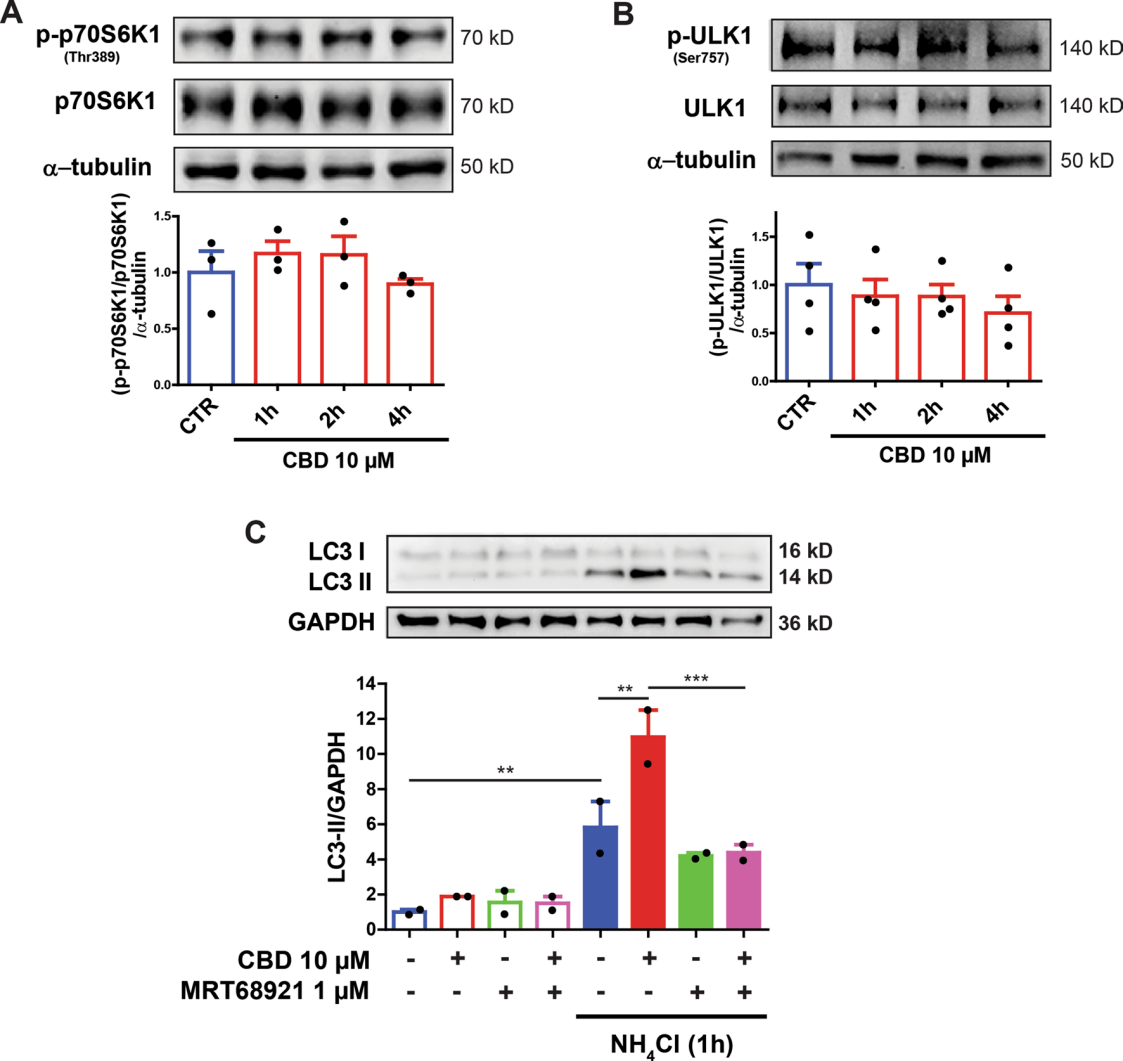
Role of mTORC1/ULK1 pathway on autophagy induction by CBD. (**A**,**B**) Total lysates from SH-SY5Y cells were treated with 10 µM CBD for 1, 2 and 4 h. The blots were incubated with a specific antibody for phosphorylated p70S6K1 (Thr389) and ULK1 (Ser757) or with an antibody that recognizes total p70S6K1 and ULK1. No effect was observed on the mTOR targets (**A**) p70S6K1 (Thr389) and (**B**) ULK1 (Ser757) after treatment with CBD, thus indicating that autophagy activation is independent of mTORC1. Data are expressed as means ± S.E.M., and data points are displayed as dots. The analysis was conducted with a one-way ANOVA followed by Dunnett’s post-hoc test. *p < 0.05, ** *p* < 0.01 relative to respective CTR group. (**C**) The autophagic flux was evaluated in SH-SY5Y cells pretreated with 1 µM of the ULK1/2 inhibitor MRT68921 for 30 min) and treated with 10 µM CBD for 2 h, in the presence or absence of the lysosome inhibitor NH_4_Cl, at the last hour of the treatment. The 10 µM CBD treatment increased LC3-II levels under NH_4_Cl blockade, which was abolished MRT6892 pre-treatment. Samples were subjected to western blotting using anti-LC3 and anti-GAPDH antibodies. Representative images of LC3-II are shown above the bar graphs. The bar graphs are reported as the means ± S.E.M of LC3-II levels after GAPDH normalization. Analysis was conducted with Three-way ANOVA followed by Sidak’s post-hoc test; *p < 0.05, ** *p* < 0.01 and ***p < 0.001. Full-length blots are presented in Supplementary Figure S4.

To further clarify that the ERK1/2 and AKT pathways were modulating mammalian target of rapamycin complex 1 (mTORC1) and Unc51-like kinase (ULK1) signaling via coordinated mechanisms, we evaluated downstream regulators of mTORC1, including the phosphorylation levels of p70 ribosomal protein S6 kinase β 1 (p70S6K1; Thr389) and ULK1 (Ser757) following CBD treatment for 1, 2 and 4 h. As shown in Fig. 5 A and B, CBD did not significantly alter p70S6K1 [*F*_3,12_ = 0.883; *p* = 0.490] or ULK1 [*F*_3,12_ = 0.054; *p* = 0.982] phosphorylation levels. Therefore, CBD-induced autophagy activation proceeds through an mTORC1-independent mechanism. Interestingly, pre-treating the cells with 1 µM MRT68921, a potent and selective ULK1/2 inhibitor^47^, for 30 min, treating the cells with CBD and blocking autophagy in the final one hour of treatment, abolished the CBD-induced increase in LC3-II under the same conditions (Fig. 5C) [three-way ANOVA: *F*_1,8_ = 6.456; p = 0.035]. Thus, CBD-induced autophagy is dependent on the ULK1 pathway.

**Figure 6:**
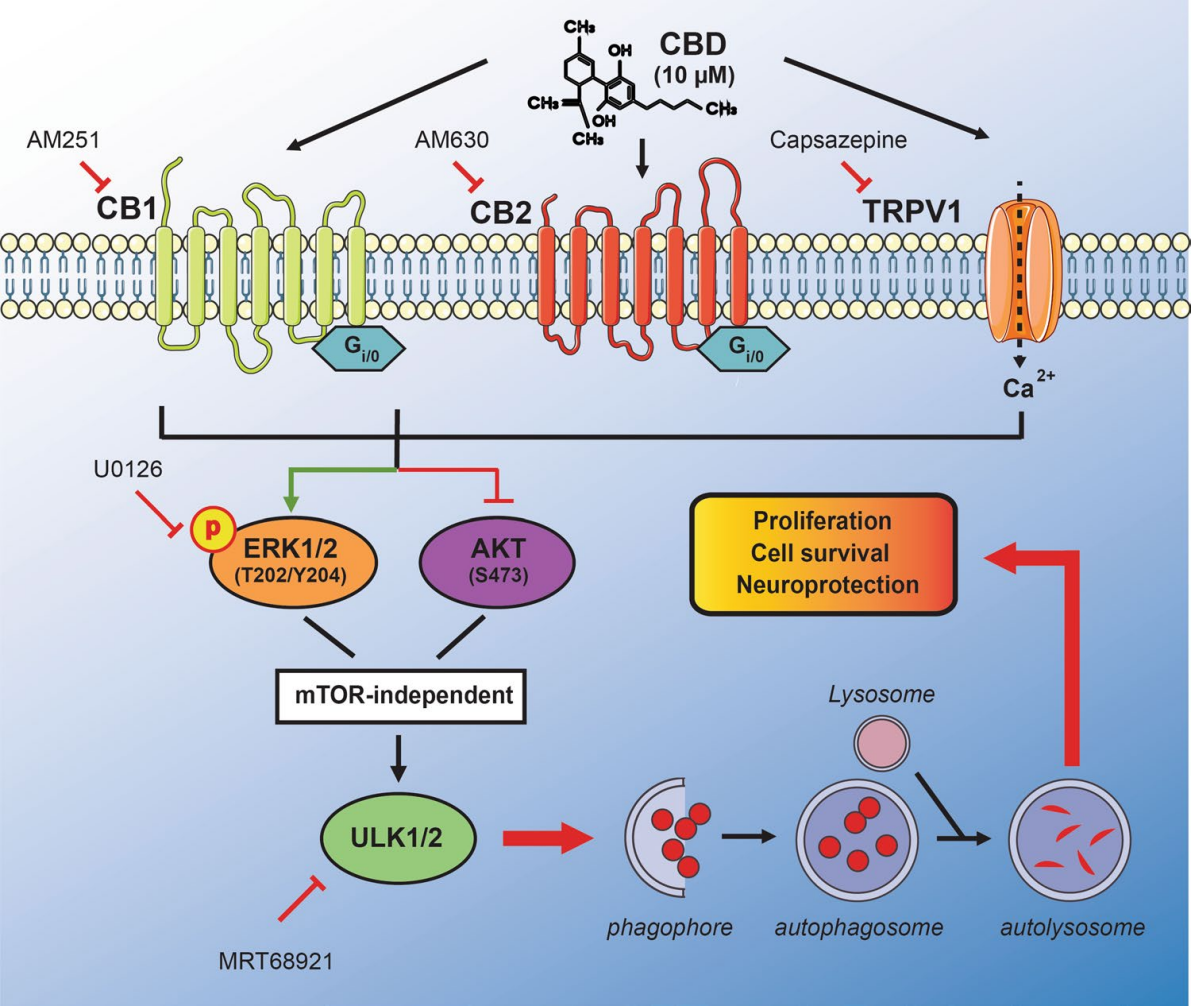
Schematic representation of the signaling pathway mediated by CBD in SH-SY5Y cells. At 10 µM, CBD activates autophagy, resulting in the accumulation of LC3-II and increased presence of LC3-positive puncta. AM 251, AM 630 and CPZ antagonists abrogated the observed LC3-II accumulation, thus implicating canonical cannabinoids (CB1 and CB2) and TRPV1 receptors in CBD-mediated autophagy. Additionally, CBD activated the ERK1/2 signaling cascade by increasing phosphorylation at Tyr204/Thr202 after 10 min of CBD treatment but suppressed the PI3K/AKT pathway by reducing AKT phosphorylation at S473. On the other hand, CBD treatment did not change AMPK phosphorylation at Thr172 or the phosphorylation levels of autophagy-related proteins ULK1 (Ser757) and p70S6K (Thr389). In conclusion, our findings suggest that CBD-induced autophagy activation is dependent on ULK1 and occurs in an mTORC1 independent-manner. Furthermore, the effects on the ERK1/2 and PI3K/AKT pathways, which regulate cell survival and proliferation, indicate that CBD could elicit neuroprotective actions. The illustration was produced using smart servier medical art vectors for publications and presentations licensed under the Creative Commons (CC BY 3.0) available at smart.servier.com.

Our findings collectively reveal a possible non-canonical autophagy activation mechanism in neural cells that involves crosstalk between the upstream regulators ERK1/2, AKT kinase, and downstream ULK1 signaling, which is not dependent on mTORC1 activity. Furthermore, the present study highlights the therapeutic potential of cannabinoid treatment for neurodegenerative disorders (Fig. 6).

## Discussion

Studies investigating the utility and efficacy of CBD in a variety of conditions and diseases have been gaining momentum in the last decades. Much of this increase in interest is due to the broad spectrum of potential therapeutic benefits and lack of psychotomimetic effects^1^. Recent studies have reported that cannabinoid-induced autophagy in cancerous and non-cancerous cells can elicit either protective or harmful effects^48,49^. Herein we sought to evaluate the mechanism by which CBD regulates autophagy, an essential protein-recycling pathway in neural cells.

In our study, 10 µM CBD modulated the autophagic flux without inducing cytotoxicity^41,50^. We focused on CBD nontoxic concentrations to evaluate the autophagic flux and compared unstimulated cells and CBD-stimulated cells with different concentrations in the presence, or absence, of the lysosomal inhibitor NH_4_Cl according to Autophagy Guidelines^51^. In order to discriminate the increased autophagosome formation promoted by the CBD treatment, the inhibition of lysosomal activity was necessary to lead the “undigested” lipidated LC3-II to accumulation. Under NH_4_Cl blockade, LC3-II is available to be analyzed by comparing the levels in the untreated cells with respect to the one treated with lysosomal inhibitors (*e.g*. NH_4_Cl) to measure the rate of autophagosome degradation defined as autophagic flux^51^. Interestingly, the NH_4_Cl treatment induced a further increased LC3-II in CBD-treated cells to higher levels when compared to control cells, indicating that LC3-II formation are not due to inhibition of autophagic machinery but due to enhanced autophagy progression that can lead to faster LC3-II degradation, as observed in control cells. Therefore, based on the results obtained, we believe that the increase in autophagic flux mediated by CBD alone was not observed due to the high degradation of LC3-II without the lysosomal inhibitor, NH_4_Cl. However, using the autophagy inhibitor we are able to observe the increase in autophagosomes (LC3-II formation) mediated by CBD.

This observation indicates that a specific concentration of CBD can activate autophagy and protect cells from potentially damaging conditions. Such effects are particularly relevant to neurodegenerative diseases since the autophagy-mediated degradation of misfolded proteins and protein aggregates is necessary for cellular homeostasis and survival^52,53^. As shown by Casarejos et al. (2013), Sativex, a drug that contains Δ^9^-THC and CBD, can increase autophagy, consequently reducing tau and amyloid proteins deposition in a tauopathy mouse model^54^. Furthermore, other studies have reported that CBD increases LC3-II levels in human colonic epithelial cells, breast cancer cells (MDA-MB-231) and in glioblastoma multiform (GBM) cell line^31,40,50^, similar to the results presented here. Despite these promising results, the pathophysiological circumstances and the underlying CBD-mediated autophagy activation mechanisms must be thoroughly considered when deciding whether CBD should be administered or not and will require further investigation.

Besides the autophagy-related effects, cannabinoids have also been shown to modulate intracellular signaling pathways involved in neuronal survival and apoptosis^55,56^. In this sense, it is essential to determine if the cannabinoid induces autophagy and elucidate the mechanism(s) of action and pathological processes to determine the therapeutic benefits they can evoke^57,58^. Indeed, it is for this reason that we also examined the activation pathways associated with the CBD receptors.

For example, using CB1 and CB2 antagonists, AM 251 and AM 630, respectively^40,59–61^, as well as the capsazepine, the vanilloid receptor antagonist, which is widely used to inhibit TRPV1 receptors^40,62^, we found that all of the employed antagonists tested attenuated CBD-induced autophagy in both SH-SY5Y cells and astrocytes. These results strongly indicate that the activation of these three CBD-sensitive receptors is involved in autophagosome formation and degradation. While identifying these receptors as active participants in the observed CBD-induced autophagy provided valuable insights into the mechanism of action, it was still necessary to assess the effects CBD elicits after binding the receptors.

The PI3K/AKT and MAPK/ERK pathways are considered crucial regulators of cell metabolism, growth, proliferation^35,36^ and vital to autophagy signal transduction^44,45^. Here we showed that CBD could activate autophagy by stimulating ERK1/2 and inhibit AKT signaling via the differential phosphorylation of these proteins. It is worth mentioning that CBD did not affect AMPK phosphorylation and, consequently, its activity, which further confirms the crucial role of the crosstalk between the autophagy-related kinases, ERK1/2 and AKT. Indeed, the regulation of PI3K/AKT is negatively modulated by MAPK/ERK activation. This conclusion is in line with a previous study that showed the inhibition of ERK1/2 signaling, using U0126, increased AKT phosphorylation at Thr308 and Ser473, and the activation of the ERK/MAPK cascade attenuated growth factor-induced AKT phosphorylation^63^. Furthermore, another study reported that U0126-mediated inhibition of ERK1/2 signaling blocks the proliferation of neural progenitor cells^64^.

Since ERK1/2 activation has been associated with cannabinoid receptors^65^, it is plausible that CB1 activation by cannabinoids is involved in neuronal protection and proliferation^66^. In this context, the link between CBD-mediated autophagy and AKT is relevant to neurodegenerative diseases. For example, 10 µM CBD exerted protective effects via AKT reduction in an SH-SY5Y cell model of Parkinson's disease induced by the toxin 1-methyl-4-phenylpyridinium (MPP^+^)^67^. Indeed, CB1^68^ or TRPV1 receptors^69^ have been shown to differentially modulate the PI3K/AKT pathways, depending on the cell type, treatment or injury^70,71^.

It has been demonstrated that mTORC1 activity negatively regulates the ULK1 complex, which is required for autophagy initiation^72^. For this purpose, we investigated if the CBD-induced autophagy would be dependent on ULK1 pathway. Pre-treatment with MRT68921, a potent and selective ULK1/2 inhibitor^47^, abolished the further increase in LC3-II observed in the presence of CBD. Collectively, our findings suggest a cross talk between the upstream regulators ERK1/2 and AKT kinase through ULK1 signaling, but not dependent on mTORC1 activity. Here, we found that CBD activated the autophagic flux in an mTORC1 independent-manner since its protein targets, p70S6K1 (Thr389) and ULK1 (Ser757), were not modulated upon CBD treatment. These findings are consistent with the study of Hiebel et al. that demonstrated CB1 receptors could modulate autophagy activation in an mTORC1 and Beclin-1 independent manner^73^. In contrast, here we report that CBD-mediated autophagy depends on the proautophagic factor ULK1, suggesting a novel noncanonical autophagy signaling pathway involving direct cross talk by ERK1/2 and PI3K/AKT. These upstream autophagic-related kinases have a putative role in linking growth factor signals and mTORC1 activity^74^.

Concerning AKT, once activated, it phosphorylates and inactivates the tuberous sclerosis complex (TSC1/2 complex), a critical negative regulator of mTORC1 activity^74^. In contrast, CBD inhibits AKT but not mTORC1. Additionally, ERK1/2 inhibits TSC2 in response to growth factor^75^. Herein, we found that CBD inhibits AKT and activates ERK1/2; hence, it is likely that ERK1/2 would inhibit TSC2, resulting in unaltered mTORC1 and stimulated ULK1 activity. This possible mechanism was previously reported in a study showing that trehalose promoted cell clearance in neurodegenerative storage diseases models^76^. While the present study revealed novel therapeutic targets for the treatment of neurodegenerative processes, future studies will need to be carried out to understand the exact mechanisms related to regulating autophagosome biogenesis.

## Conclusions

As depicted in Fig. 6, the present study demonstrated that CBD plays an important role in autophagy activation by regulating the phosphorylation of ERK1/2 and AKT kinases with involvement of ULK1, but in an mTORC1-independent pathway. The activation of these signaling pathways involves the cannabinoid receptors, CB1 and CB2, as well as TRPV1 receptors, which are essential for promoting neuronal cell survival and proliferation. This study not only provides details about the underlying mechanisms involved in cannabinoid-mediated autophagy activation but also highlights the promising potential of cannabidiol therapy for neurodegenerative disorders.

## Material and methods

### Drugs

Cannabidiol (CBD) was purchased from BSPG—Pharm (Sandwich, Kent, UK), N-(Piperidin-1-yl)-5-(4-iodophenyl)-1-(2,4-dichlorophenyl)-4-methyl-1H-pyrazole-3-carboxamide (AM 251), 6-Iodo-2-methyl-1-[2-(4-morpholinyl)ethyl]-1H-indol-3-yl](4-methoxyphenyl)methanone (AM 630), capsazepine (CPZ) and Earle's Balanced Salt Solution (EBSS) were purchased from Sigma-Aldrich Chemical Co. (St Louis, MO, USA), N-[3-[[5-Cyclopropyl-2-[(1,2,3,4-tetrahydro-2-methyl-6-isoquinolinyl)amino]-4-pyrimidinyl]amino]propyl]-cyclobutanecarboxamide dihydrochloride (MRT68921) was purchased from Cayman Chemical (Michigan, USA) and 1,4-Diamino-2,3-dicyano-1,4-bis[2-aminophenylthio]butadiene (U0126) was purchased from Cell Signaling Technology (Beverly, MA, USA).

### Cell culture

The human neuroblastoma (SH-SY5Y-ATCC-CRL-2266) and murine immortalized astrocyte^77^ cell lines were cultured in high-glucose Dulbecco's Modified Eagle Medium, supplemented with 10% fetal bovine serum and 1% penicillin/streptomycin (Thermo Fisher Scientific, Waltham, MA, USA) and maintained at 37°C in a 5% CO_2_ atmosphere^78^. These neural cell lines have been extensively used in in vitro studies involving cell death mechanisms and express functional CB1, CB2 and TRPV1 receptors^79,80^. The cells were transferred to 6-well plates (5 × 10^5^ cells/well) for the flow cytometry, immunoblotting and immunofluorescence assays (Thermo Fisher Scientific). The Institutional Ethics Committee of the Federal University of São Paulo (UNIFESP) approved all of the experimental protocols and procedures (Protocol number 5810061017).

### Cell death measurements

Cells were treated with seven concentrations of CBD (1, 2, 5, 10, 25, 50, 100 µM). As a positive control, 1 µM staurosporine (STS), a nonselective protein kinase inhibitor that leads cells to apoptosis^81^, was used. The cells were fixed using cold ethanol (50% in PBS) after 24 or 48 h of treatment. The fixed cells were then pelleted and stained with 25 μg/mL Propidium Iodide (PI) in PBS. Flow cytometry experiments to calculate the percentage of cells in the sub-G_0_/G_1_ phase were performed with the BD FACSCalibur platform (Becton–Dickinson, Mountain View, CA, USA), and the data were acquired with the FL2 channel considering 10,000 events. The results were analyzed using the FlowJo Software (BD Biosciences, Franklin Lakes, NJ, USA).

### Western blotting analysis

Cells were treated according to each experimental goal and lysed in RIPA buffer (150 mM NaCl, 1% NP-40, 0.5%, deoxycholic acid, 0.1% SDS, 50 mM Tris pH 8.0, and 2 mM MgCl_2_)_._ Protease and phosphatase inhibitors (1:100 protease inhibitor cocktail plus, 10 mM sodium fluoride, 1 mM sodium orthovanadate, 1 mM sodium molybdate and PMSF; Sigma-Aldrich) were included during cell lysis. Samples were incubated at 4 °C for 30 min and centrifuged at 4 °C for 10 min (13,000 rpm) to remove insoluble debris. Protein concentrations were determined with the Bradford assay (Bio-rad, Hercules, CA, USA), and standard mini gels were loaded with 15–30 µg of total proteins and subjected to SDS-PAGE. The SDS-PAGE gel contents were then transferred to nitrocellulose or PVDF membranes (Millipore, Massachusetts, USA) using a Trans-Blot cell system (Bio-rad). Membranes were incubated with the primary antibodies in PBS containing 5% non-fat dry milk and 0.1% Tween-20 overnight at 4 °C. The primary antibodies utilized for this study included anti-LC3B (#2775S, 1:2000), anti-p-p44/42 MAP kinases (Tyr204/Thr202, #91015, 1:1000), anti-p44/42 MAP kinases (#91025, 1:1000), anti-p-AKT (Ser473, #9271, 1:1000), anti-AKT (#46915, 1:1000), anti-p-AMPKα (Thr172, #25315, 1:1000), anti-AMPKα (#25325, 1:1000), anti-p-p70S6K1 (Thr389, #9205, 1:1000), anti-p70S6K1 (#9202, 1:1000), anti-p-ULK1 (Ser757, #6888, 1:1000) and anti-ULK1 (#8054, 1:1000), which were all purchased from Cell Signaling Technology Inc. The density of α-tubulin or GAPDH served as loading controls in all of the experiments, using monoclonal mouse antibodies, anti-α-tubulin (Sigma-Aldrich, #T8203, 1:5000) or anti-GAPDH (Sigma-Aldrich, #G8795, 1:5000), respectively. After washing away nonbound primary antibody, the appropriate horseradish peroxidase-conjugate secondary-antibody (anti-rabbit or anti-mouse IgG; Jackson Immunoresearch) were incubated with the immunoblots at a dilution of 1:5000 for 1 h. The immunoblots were then visualized using the Western Lightning Plus-ECL chemiluminescence system (Perkin Elmer). Signals were recorded using an Uvitec chemidoc-imaging platform, and densitometry was performed with the Uvitec Alliance software. The optical densities of all the bands were normalized to the density of either the α-tubulin or GAPDH bands in each experiment.

### Retroviral transduction and confocal microscopy for autophagy evaluation

As previously described^82^, to produce the virus for this protocol, 15 µg of the pLPCX-mCherry-LC3 plasmid and 5 µg of vesicular stomatitis virus G protein expression plasmid were transfected into the 293 gp/bsr cell line using the calcium phosphate protocol^83^. After 48 h, the retroviral particles present in the supernatant were recovered, supplemented with polybrene 4 µg/mL and stored at – 80 °C until the transduction. The mCherry-LC3-overexpressing SH-SY5Y cells were plated on 13 mm glass coverslips at a density of 5 × 10^5^ cells/mL. After 24 h, the cells were treated with 10 µM CBD for 2 h. Following this treatment, cells were fixed with 4% paraformaldehyde and observed under a Zeiss LSM 780 Confocal Microscopy (Carl Zeiss, Oberkochen, Germany). Images were analyzed with the ImageJ software (NIH, Bethesda, MD, USA).

### Statistics

The Shapiro–Wilk and Levene’s tests were used to evaluate data normality and homogeneity of variance assumptions, respectively, and parametric hypothesis tests were employed according to the data distribution. Briefly, one, two, or three-way Analysis of Variance (ANOVA) was applied to experimental factors. The results were used to estimate single treatment and multi-treatment interaction effects on the observed variable. Differences between groups were detected using Sidak’s or Dunnett’s post-hoc tests when appropriate. Data are expressed as the mean ± standard error of the mean (S.E.M.), and the significance level was set at *p* < 0.05.

## Supplementary Information

Supplementary Information 1.

Supplementary Information 2.
