# Supplementary material for: Cannabidiol induces autophagy via ERK1/2 activation in neural cells
Source: Sci Rep. 2021 Mar 8;11:5434. doi: 10.1038/s41598-021-84879-2 (PMC7940388; doi:10.1038/s41598-021-84879-2)
Supplement: Supplementary file 1 — Supplementary Information 1. [file 41598_2021_84879_MOESM1_ESM.pdf]

Supplementary Table 1

Three-way ANOVA effect	*F* statistic	*df*	*p*-value
CBD *versus* AM251 *versus* NH_4Cl interaction	8.541	(1,40)	$p = 0.006$
CBD *versus* AM630 *versus* NH_4Cl interaction	3.923	(1,32)	$p = 0.047$
CBD *versus* CPZ *versus* NH_4Cl interaction	4.249	(1,40)	$p = 0.045$

Pairwise comparisons (Sidak's *post hoc* test)			
CBD versus AM251 versus NH_4Cl experiment			***p*-value**
CBD 10 µM$^{(-)}$ / AM251$^{(-)}$ / NH_4Cl	*vs*	CBD 10 µM$^{(-)}$ / AM251$^{(-)}$ / CTR	$p = 0.024$
CBD 10 µM$^{(+)}$ / AM251$^{(-)}$ / NH_4Cl	*vs*	CBD 10 µM$^{(-)}$ /AM251$^{(-)}$ / NH_4Cl	$p = 0.003$
CBD 10 µM$^{(+)}$ / AM251$^{(-)}$ / NH_4Cl	*vs*	CBD 10 µM$^{(+)}$ /AM251$^{(+)}$ / NH_4Cl	$p = 0.001$
CBD versus AM630 versus NH_4Cl experiment			***p*-value**
CBD 10 µM$^{(-)}$ / AM630$^{(-)}$ / NH_4Cl	*vs*	CBD 10 µM$^{(-)}$ / AM630$^{(-)}$/ CTR	$p = 0.018$
CBD 10 µM$^{(+)}$ / AM630$^{(-)}$ / NH_4Cl	*vs*	CBD 10 µM$^{(-)}$ / AM630$^{(-)}$ / NH_4Cl	$p < 0.001$
CBD 10 µM$^{(+)}$ / AM630$^{(-)}$ / NH_4Cl	*vs*	CBD 10 µM$^{(+)}$ / AM630$^{(+)}$ / NH_4Cl	$p < 0.001$
CBD versus CPZ versus NH_4Cl experiment			***p*-value**
CBD 10 µM$^{(-)}$ / CPZ$^{(-)}$ / NH_4Cl	*vs*	CBD 10 µM$^{(-)}$ / CPZ$^{(-)}$/ CTR	$p < 0.001$
CBD 10 µM$^{(+)}$ / CPZ$^{(-)}$ / NH_4Cl	*vs*	CBD 10 µM$^{(-)}$ / CPZ$^{(-)}$ / NH_4Cl	$p < 0.001$
CBD 10 µM$^{(+)}$ / CPZ$^{(-)}$ / NH_4Cl	*vs*	CBD 10 µM$^{(+)}$ / CPZ$^{(+)}$ / NH_4Cl	$p < 0.001$

Table S1. Statistical summary for CBD *versus* antagonists (AM251, AM630, CPZ) under the presence or not of NH_4Cl autophagic flux blockade in SH-SY5Y cells. Three-way ANOVA followed by Sidak's *post hoc* test for pairwise comparisons; respective *F* statistic, degree of freedom (*df*), and *p*-values are indicated in the table for every hypothesis test.

Supplementary Table 2

**Three-way ANOVA effect**	***F* statistic**	***df***	***p*-value**
CBD *versus* AM251 *versus* NH4Cl interaction	4,352	(1,32)	$p = 0.045$
CBD *versus* AM630 *versus* NH_4Cl interaction	6,674	(1,32)	$p = 0.015$
CBD *versus* CPZ *versus* NH_4Cl interaction	5,498	(1,32)	$p = 0.025$

**Pairwise comparisons (Sidak's *post hoc* test)**			
CBD versus AM251 versus NH_4Cl experiment			***p*-value**
CBD 10 µM$^{(-)}$ / AM251$^{(-)}$ / NH_4Cl	*vs*	CBD 10 µM$^{(-)}$ / AM251$^{(-)}$ / CTR	$p < 0.001$
CBD 10 µM$^{(+)}$ / AM251$^{(-)}$ / NH_4Cl	*vs*	CBD 10 µM$^{(-)}$ /AM251$^{(-)}$ / NH_4Cl	$p = 0.001$
CBD 10 µM$^{(+)}$ / AM251$^{(-)}$ / NH_4Cl	*vs*	CBD 10 µM$^{(+)}$ /AM251$^{(+)}$ / NH_4Cl	$p = 0.001$
CBD versus AM630 versus NH_4Cl experiment			***p*-value**
CBD 10 µM$^{(-)}$ / AM630$^{(-)}$ / NH_4Cl	*vs*	CBD 10 µM$^{(-)}$ / AM630$^{(-)}$/ CTR	$p < 0.001$
CBD 10 µM$^{(+)}$ / AM630$^{(-)}$ / NH_4Cl	*vs*	CBD 10 µM$^{(-)}$ / AM630$^{(-)}$ / NH_4Cl	$p < 0.001$
CBD 10 µM$^{(+)}$ / AM630$^{(-)}$ / NH_4Cl	*vs*	CBD 10 µM$^{(+)}$ / AM630$^{(+)}$ / NH_4Cl	$p < 0.001$
CBD versus CPZ versus NH_4Cl experiment			***p*-value**
CBD 10 µM$^{(-)}$ / CPZ$^{(-)}$ / NH_4Cl	*vs*	CBD 10 µM$^{(-)}$ / CPZ$^{(-)}$/ CTR	$p = 0.002$
CBD 10 µM$^{(+)}$ / CPZ$^{(-)}$ / NH_4Cl	*vs*	CBD 10 µM$^{(-)}$ / CPZ$^{(-)}$ / NH_4Cl	$p = 0.001$
CBD 10 µM$^{(+)}$ / CPZ$^{(-)}$ / NH_4Cl	*vs*	CBD 10 µM$^{(+)}$ / CPZ$^{(+)}$ / NH_4Cl	$p = 0.001$

Table S2. Statistical summary for CBD *versus* antagonists (AM251, AM630, CPZ) under the presence or not of NH_4Cl autophagic flux blockade in immortalized astrocytes cells. Three-way ANOVA followed by Sidak's *post hoc* test for pairwise comparisons; respective *F* statistic, degree of freedom (*df*), and *p*-values are indicated in the table for every hypothesis test.
