# Supplementary material for: Cannabidiol induces autophagy via ERK1/2 activation in neural cells
Source: Sci Rep. 2021 Mar 8;11:5434. doi: 10.1038/s41598-021-84879-2 (PMC7940388; doi:10.1038/s41598-021-84879-2)
Supplement: Supplementary file 2 — Supplementary Information 2. [file 41598_2021_84879_MOESM2_ESM.pdf]

CANNABIDIOL INDUCES AUTOPHAGY VIA ERK1/2 ACTIVATION IN NEURAL CELLS

Talita A. M. Vrechi (1), Anderson H. F. F. Leão (1), Ingrid B. M. Morais (1), Vanessa C. Abílio (1,3), Antonio W. Zuardi (4,5), Jaime Eduardo C. Hallak (4,5), José Alexandre Crippa (4,5), Claudia Bincoletto (1), Rodrigo P. Ureshino (2,3), Soraya S. Smaili (1), Gustavo J. S. Pereira (1)*

1. Department of Pharmacology, Escola Paulista de Medicina, Universidade Federal de São Paulo, São Paulo, SP, Brazil.
2. Department of Biological Sciences, Diadema Campus, Universidade Federal de São Paulo, Diadema, SP Brazil.
3. Laboratory of Molecular and Translational Endocrinology, Escola Paulista de Medicina, Universidade Federal de São Paulo, São Paulo, SP Brazil.
4. National Institute for Translational Medicine (INCT-TM, CNPq), Ribeirão Preto, Brazil.
5. Department of Neuroscience and Behavior, Universidade de São Paulo, USP, Ribeirão Preto, Brazil.

Running title: Cannabidiol induces autophagy via ERK1/2 activation.

*Corresponding author: Gustavo J. S. Pereira, Department of Pharmacology, Escola Paulista de Medicina, Universidade Federal de São Paulo, Três de Maio street, 100, Zip Code: 04044-020, São Paulo, SP, Brazil. Tel: +55-11-5576-4449; e-mail: gustavo.pereira@unifesp.br.

Supplementary material

Supplementary Table 1

**Three-way ANOVA effect**	***F* statistic**	***df***	***p*-value**
CBD *versus* AM251 *versus* NH_4Cl interaction	8.541	(1,40)	$p = 0.006$
CBD *versus* AM630 *versus* NH_4Cl interaction	3.923	(1,32)	$p = 0.047$
CBD *versus* CPZ *versus* NH_4Cl interaction	4.249	(1,40)	$p = 0.045$

**Pairwise comparisons (Sidak's *post hoc* test)**			
CBD versus AM251 versus NH_4Cl experiment			***p*-value**
CBD 10 μM$^{(-)}$ / AM251$^{(-)}$ / NH_4Cl	*vs*	CBD 10 μM$^{(-)}$ / AM251$^{(-)}$ / CTR	$p = 0.024$
CBD 10 μM$^{(+)}$ / AM251$^{(-)}$ / NH_4Cl	*vs*	CBD 10 μM$^{(-)}$ /AM251$^{(-)}$ / NH_4Cl	$p = 0.003$
CBD 10 μM$^{(+)}$ / AM251$^{(-)}$ / NH_4Cl	*vs*	CBD 10 μM$^{(+)}$ /AM251$^{(+)}$ / NH_4Cl	$p = 0.001$
CBD versus AM630 versus NH_4Cl experiment			***p*-value**
CBD 10 μM$^{(-)}$ / AM630$^{(-)}$ / NH_4Cl	*vs*	CBD 10 μM$^{(-)}$ / AM630$^{(-)}$/ CTR	$p = 0.018$
CBD 10 μM$^{(+)}$ / AM630$^{(-)}$ / NH_4Cl	*vs*	CBD 10 μM$^{(-)}$ / AM630$^{(-)}$ / NH_4Cl	$p < 0.001$
CBD 10 μM$^{(+)}$ / AM630$^{(-)}$ / NH_4Cl	*vs*	CBD 10 μM$^{(+)}$ / AM630$^{(+)}$ / NH_4Cl	$p < 0.001$
CBD versus CPZ versus NH_4Cl experiment			***p*-value**
CBD 10 μM$^{(-)}$ / CPZ$^{(-)}$ / NH_4Cl	*vs*	CBD 10 μM$^{(-)}$ / CPZ$^{(-)}$/ CTR	$p < 0.001$
CBD 10 μM$^{(+)}$ / CPZ$^{(-)}$ / NH_4Cl	*vs*	CBD 10 μM$^{(-)}$ / CPZ$^{(-)}$ / NH_4Cl	$p < 0.001$
CBD 10 μM$^{(+)}$ / CPZ$^{(-)}$ / NH_4Cl	*vs*	CBD 10 μM$^{(+)}$ / CPZ$^{(+)}$ / NH_4Cl	$p < 0.001$

Table S1. Statistical summary for CBD *versus* antagonists (AM251, AM630, CPZ) under the presence or not of NH_4Cl autophagic flux blockade in SH-SY5Y cells. Three-way ANOVA followed by Sidak's *post hoc* test for pairwise comparisons; respective *F* statistic, degree of freedom (*df*), and *p*-values are indicated in the table for every hypothesis test.

Supplementary Table 2

Three-way ANOVA effect	*F* statistic	*df*	*p*-value
CBD *versus* AM251 *versus* NH4Cl interaction	4,352	(1,32)	$p = 0.045$
CBD *versus* AM630 *versus* NH_4Cl interaction	6,674	(1,32)	$p = 0.015$
CBD *versus* CPZ *versus* NH_4Cl interaction	5,498	(1,32)	$p = 0.025$

Pairwise comparisons (Sidak's *post hoc* test)			
CBD versus AM251 versus NH_4Cl experiment			***p*-value**
CBD 10 µM$^{(-)}$ / AM251$^{(-)}$ / NH_4Cl	*vs*	CBD 10 µM$^{(-)}$ / AM251$^{(-)}$ / CTR	$p < 0.001$
CBD 10 µM$^{(+)}$ / AM251$^{(-)}$ / NH_4Cl	*vs*	CBD 10 µM$^{(-)}$ /AM251$^{(-)}$ / NH_4Cl	$p = 0.001$
CBD 10 µM$^{(+)}$ / AM251$^{(-)}$ / NH_4Cl	*vs*	CBD 10 µM$^{(+)}$ /AM251$^{(+)}$ / NH_4Cl	$p = 0.001$
CBD versus AM630 versus NH_4Cl experiment			***p*-value**
CBD 10 µM$^{(-)}$ / AM630$^{(-)}$ / NH_4Cl	*vs*	CBD 10 µM$^{(-)}$ / AM630$^{(-)}$/ CTR	$p < 0.001$
CBD 10 µM$^{(+)}$ / AM630$^{(-)}$ / NH_4Cl	*vs*	CBD 10 µM$^{(-)}$ / AM630$^{(-)}$ / NH_4Cl	$p < 0.001$
CBD 10 µM$^{(+)}$ / AM630$^{(-)}$ / NH_4Cl	*vs*	CBD 10 µM$^{(+)}$ / AM630$^{(+)}$ / NH_4Cl	$p < 0.001$
CBD versus CPZ versus NH_4Cl experiment			***p*-value**
CBD 10 µM$^{(-)}$ / CPZ$^{(-)}$ / NH_4Cl	*vs*	CBD 10 µM$^{(-)}$ / CPZ$^{(-)}$/ CTR	$p = 0.002$
CBD 10 µM$^{(+)}$ / CPZ$^{(-)}$ / NH_4Cl	*vs*	CBD 10 µM$^{(-)}$ / CPZ$^{(-)}$ / NH_4Cl	$p = 0.001$
CBD 10 µM$^{(+)}$ / CPZ$^{(-)}$ / NH_4Cl	*vs*	CBD 10 µM$^{(+)}$ / CPZ$^{(+)}$ / NH_4Cl	$p = 0.001$

Table S2. Statistical summary for CBD *versus* antagonists (AM251, AM630, CPZ) under the presence or not of NH_4Cl autophagic flux blockade in immortalized astrocytes cells. Three-way ANOVA followed by Sidak's *post hoc* test for pairwise comparisons; respective *F* statistic, degree of freedom (*df*), and *p*-values are indicated in the table for every hypothesis test.

Figure S1

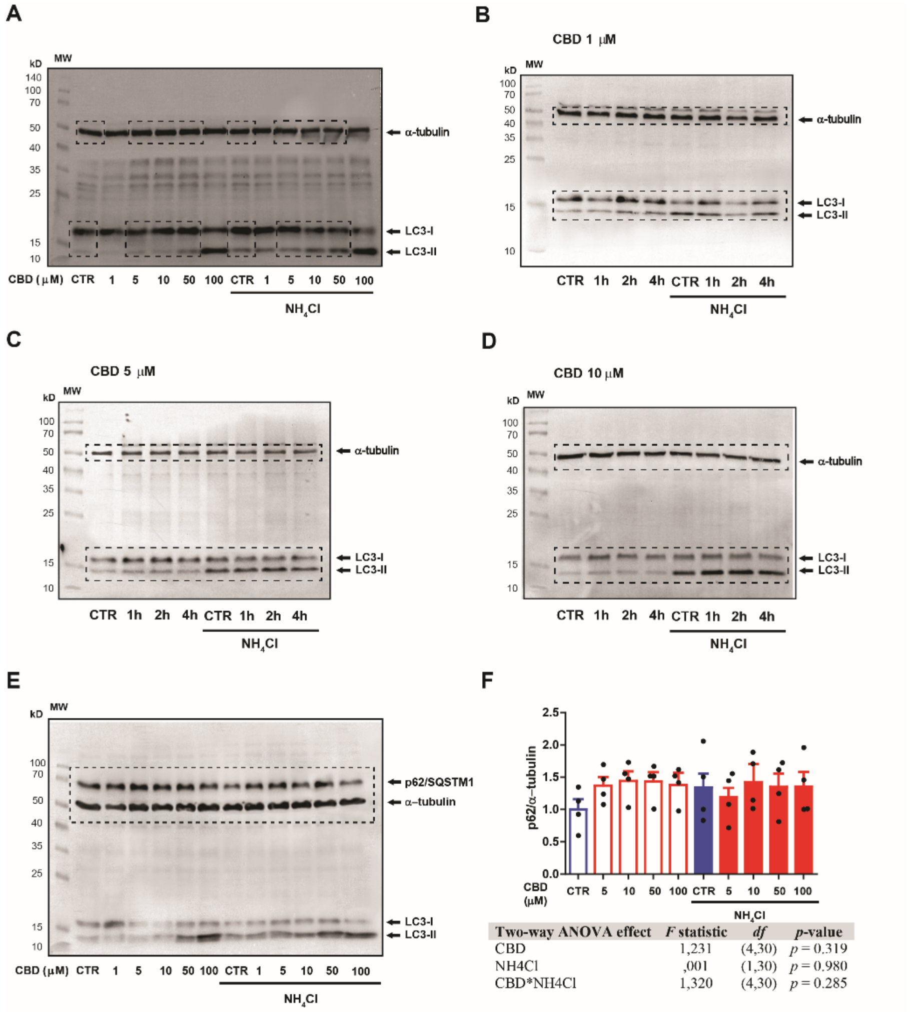

Two-way ANOVA effect	*F* statistic	*df*	*p*-value
CBD	1,231	(4,30)	*p* = 0.319
NH4Cl	,001	(1,30)	*p* = 0.980
CBD*NH4Cl	1,320	(4,30)	*p* = 0.285

Figure S1. (A-D**)** Full scans of originals Western blots for data Figure 2. The dashed lines on the panels correspond to the figures in the paper. (E) Representative blot and (F) p62/SQSTM1 expression under different concentrations of CBD. p62/SQSTM1 expression was not modulated either by CBD or NH_4Cl treatment.

Figure S2

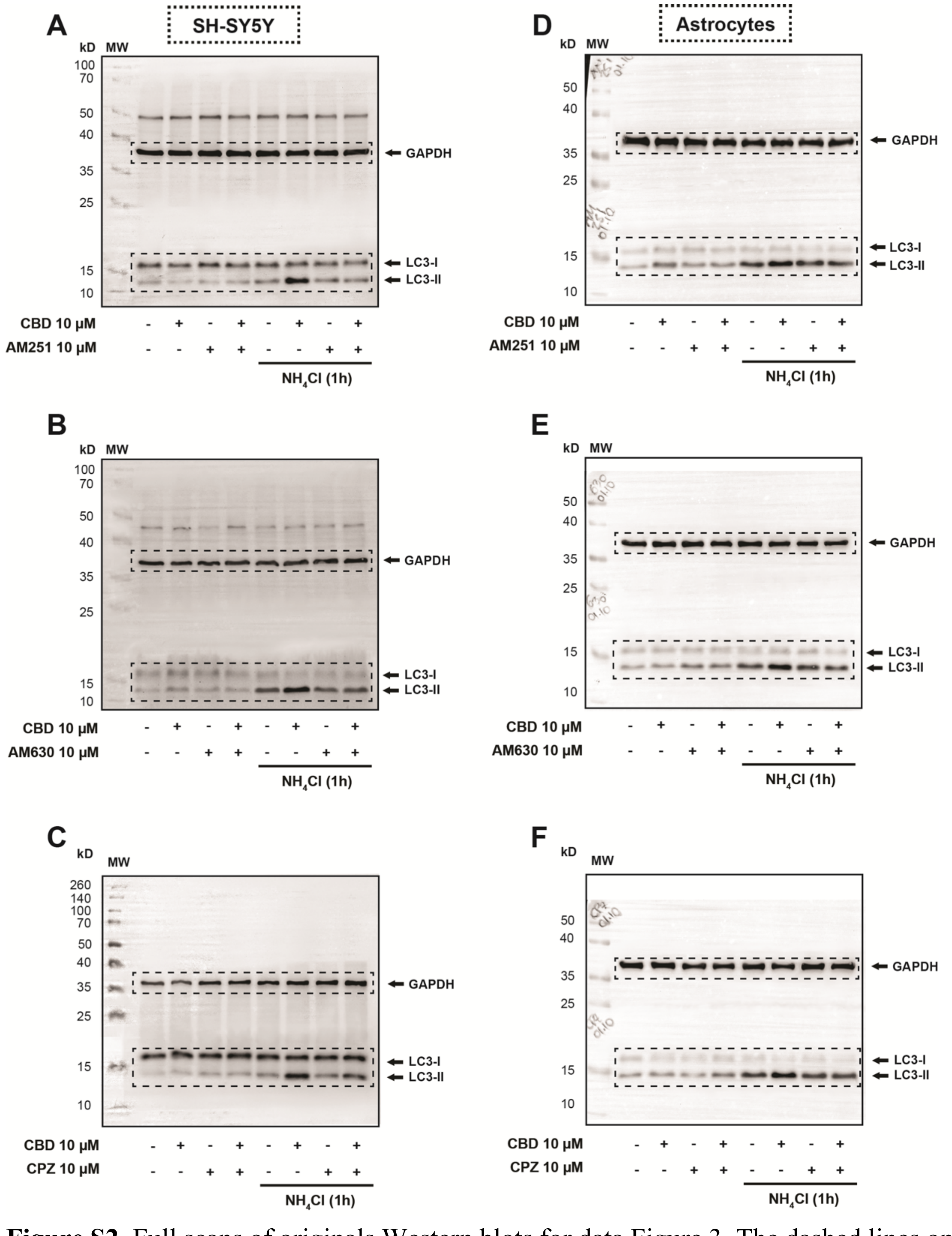

Figure S2. Full scans of originals Western blots for data Figure 3. The dashed lines on the panels correspond to the figures in the paper.

Figure S3

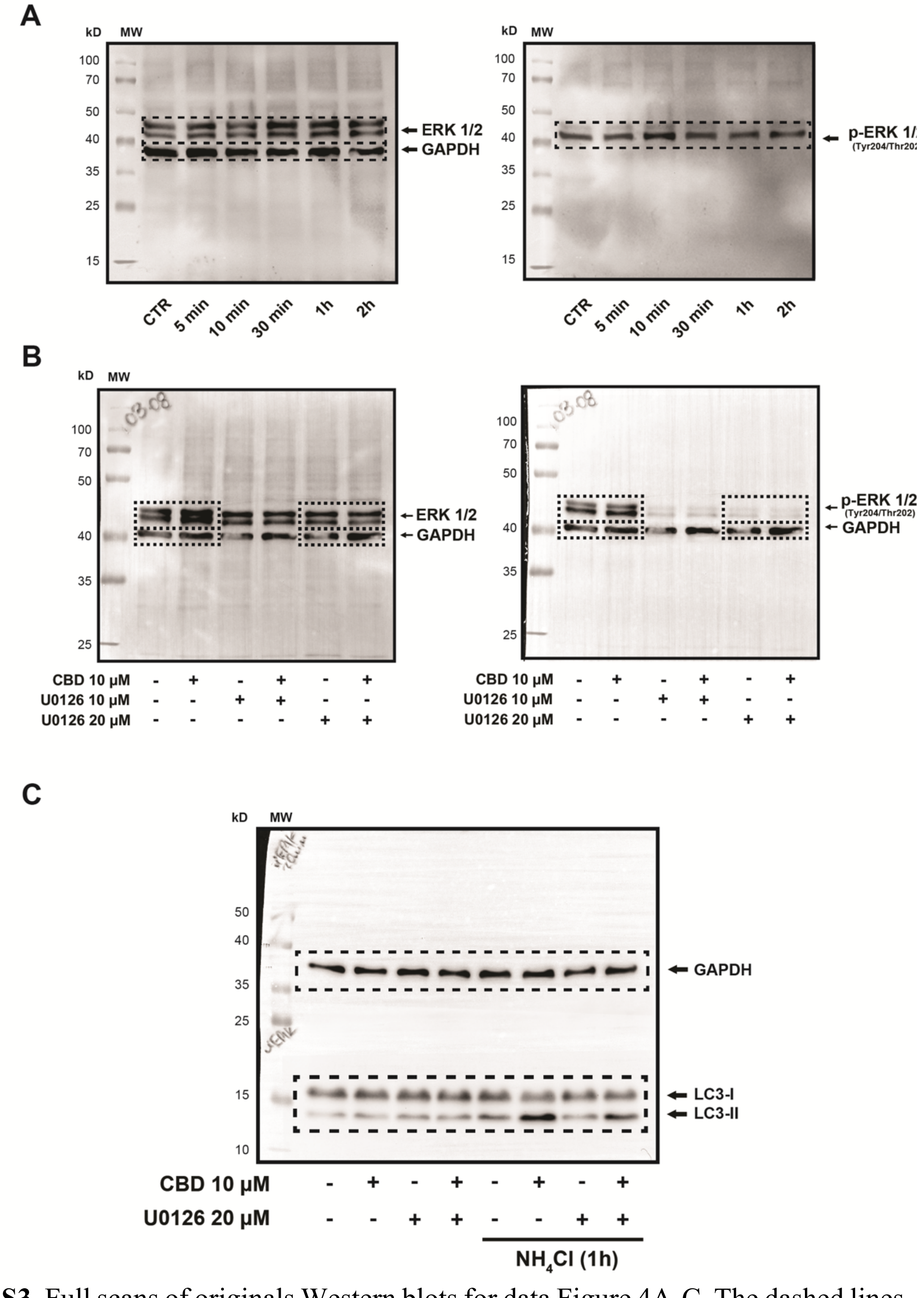

Figure S3. Full scans of originals Western blots for data Figure 4A-C. The dashed lines on the panels correspond to the figures in the paper.

Figure S4

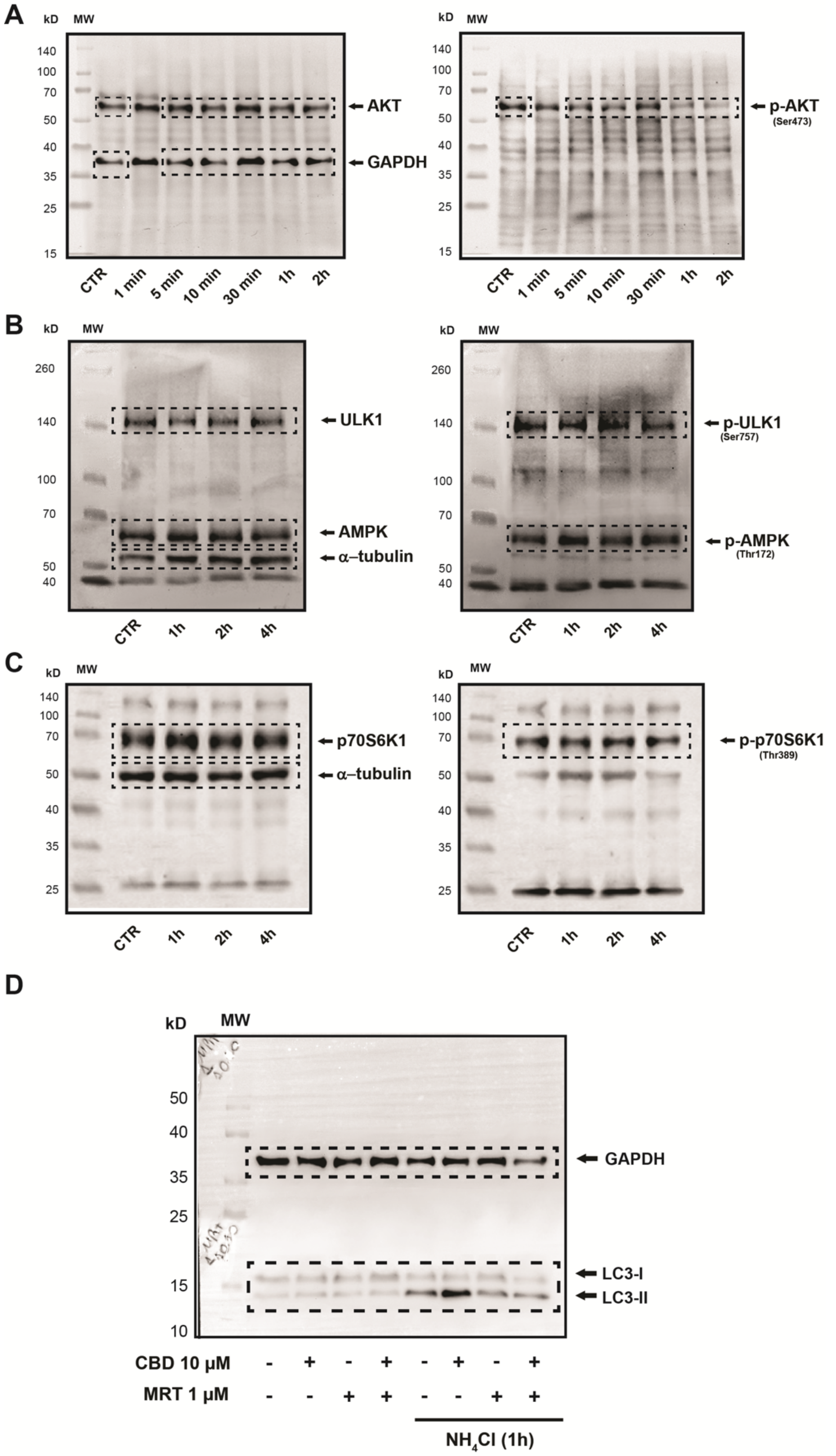

Figure S4 Full scans of originals Western blots for data Figure 4D-E and 5. The dashed lines on the panels correspond to the figures in the paper.
